# Assessment of Multicolor Flow Cytometry Panels to Study Leukocyte Subset Alterations in Water Buffalo (*Bubalus bubalis*) During BVDV Acute Infection

**DOI:** 10.3389/fvets.2020.574434

**Published:** 2020-10-16

**Authors:** Francesco Grandoni, Alessandra Martucciello, Stefano Petrini, Roberto Steri, Anna Donniacuo, Cristina Casciari, Maria Carmela Scatà, Carlo Grassi, Domenico Vecchio, Francesco Feliziani, Giovanna De Matteis, William C. Davis, Esterina De Carlo

**Affiliations:** ^1^CREA—Consiglio per la Ricerca in Agricoltura e l'Analisi dell'Economia Agraria, Centro di ricerca Zootecnia e Acquacoltura (Research Centre for Animal Production and Aquaculture), Monterotondo, Italy; ^2^Istituto Zooprofilattico Sperimentale del Mezzogiorno, National Reference Centre for Hygiene and Technologies of Water Buffalo Farming and Productions, Salerno, Italy; ^3^Istituto Zooprofilattico Sperimentale Dell'Umbria e delle Marche, Perugia, Italy; ^4^Department of Veterinary Microbiology and Pathology, Washington State University, Pullman, WA, United States

**Keywords:** flow cytometry, monoclonal antibody, water buffalo (*Bubalus bubalis*), bovine viral diarrhoea virus (BVDV), experimental infection

## Abstract

The identification of cross-reactive monoclonal antibodies (mAbs) that recognize orthologous leukocyte differentiation molecules (LDM) in buffaloes has overcome a major impediment limiting research on the immune response to pathogens and development of vaccines. As reported, two pilot trials were conducted to accomplish two objectives: (1) demonstrate that multiparameter flow cytometry can be conducted equally well in buffalo with mAbs directly and indirectly labeled with fluorochromes in research and (2) flow cytometry can be used to compare and extend studies on diseases of economic importance to buffalo using bovine viral diarrhea virus (BVDV) as a model pathogen. Pregnant buffalo cows were infected with BVDV-1 at 81 (trial 1) and 203 (trial 2) days post artificial insemination and flow cytometric evaluations were performed at 0, 3, 4, and 14 days after infection (dpi). Fluorochrome conjugated mAbs were used in trial 1, and fluorochrome conjugated goat isotype specific anti-mouse antibodies were used to label mAbs in trial 2. Flow cytometric analysis revealed a transient lymphopenia occurs during the 1st days following infection similar to lymphopenia reported in cattle. In particular, significant differences were observed between pre- and post-infection absolute values of T lymphocytes (−56%, *P* < 0.01). CD21^+^ B lymphocytes (−65%, *P* = 0.04), and Natural Killer cells (−72%, *P* < 0.001). No significant differences were observed in monocytes and neutrophil absolute values, or the CD4:CD8 ratio. Animal health status was followed until 15 days after calving. No clinical signs of infection were observed during the evaluation period, however, animals in trial 1 developed complications later the infection. One cow aborted at 57 days post-infection, the second cow developed a prolapse a day after calving and died. These two animals also showed a more pronounced lymphopenia in comparison with animals infected at 203 days of pregnancy (e.g., −77 vs. −22% T lymphocytes at 3 dpi, respectively). The pilot studies have demonstrated that it is possible to use multicolour multiparameter flow cytometry to study the immune response to pathogens affecting the health of buffalo.

## Introduction

Flow cytometry (FCM) is an important and versatile technology: flexibility, accuracy and multiparametric analysis are some of its characteristics. It has proven useful in research in microbiology, oncology, immunology, hematology, and nanotechnology. Flow cytometry was introduced into veterinary sciences in the 80's concurrent with pioneering studies to develop monoclonal antibodies (mAbs) against leukocyte differentiation molecules (LDM) in cattle and other livestock species (1–3). During this same timeframe studies were initiated to make use of the first characterized mAbs to determine the frequency of leukocyte subsets in blood and milk (4, 5). Studies were also initiated to determine if flow cytometry could be used to study sequential changes in the frequency of leukocyte subsets associated with pathogenesis of livestock pathogens starting with a persistent pathogen, BVDV in cattle (6). Excluding cattle, the use of flow cytometry in studies with other veterinary species has been constrained, attributed to the limited availability and cost of developing mAb reagents for research (7). This has included research with water buffalo. Although buffaloes are an important livestock species, to date, no resources have been available to develop mAbs against buffalo leukocyte differentiation molecules (LDM) for use in the study of the immunopathogenesis of pathogens and development of vaccines. Fortunately, it has been possible to take advantage of the phylogenetic relation of buffalo with cattle to circumvent this constraint. The antigenic composition of many of the molecules expressed on leukocytes have been conserved. Screening of mAbs developed against LDM in cattle, sheep, and goat revealed a large set of the mAbs recognize epitopes conserved on orthologous LDM in buffalo (8–11). The set of cross-reactive mAbs now make it possible to conduct studies on the immune response to pathogens equivalent to ongoing studies in cattle. The present study was undertaken to introduce the use of the mAbs in flow cytometry to conduct studies on the immunopathogenesis of pathogens in buffalo, using BVDV as a model of a pathogen with respect to which further research is needed to fully understand the mechanisms of pathogenesis. BVDV is a member of the *Flaviviridae* family, genus *Pestivirus* (12). The International Committee on Taxonomy of Viruses (ICTV) recognizes two established species namely bovine viral diarrhea virus type 1 (BVDV-1) and type 2 (BVDV-2), and a third putative bovine species, referred to as HoBi-like pestivirus (13). This is one of the groups of viral pathogens affecting livestock. Studies in cattle have revealed exposure usually leads to development of mild disease characterized by pyrexia, anorexia, leukopenia, and mild diarrhea. However, infection can also lead to development of mucosal disease and reproductive problems (abortion, teratogenesis, embryo resorption, fetal mummification, and stillbirth). Cows infected before 150 days of gestation can give birth to calves persistently infected with BVDV (12). The mechanism of bypassing the adaptive immune system by establishing tolerance permits the virus to be extremely successful by used an evasion strategy that still remains to be fully determined. A main problem with BVDV is that infection leads to development of an immune response that controls but does not clear infection. In spite of the presence of antibody and cell mediated immunity, the virus continues to persist and replicate in infected animals and is released into the environment. Infected cows and calves serve as reservoirs contaminating the environment. Most of the reported BVDV infections refer to infections in the *Bovidae* family. Only a few of them refer to infection in buffaloes, and most of these have been reports on seroprevalence (14–16). The presence of the BVDV-1 RNA has been demonstrated in fetal serum in buffaloes with mucosal disease in Australia (17) and Argentina (18). In Italy, viruses of sub-genotype BVDV-1a (19) and BVDV-1b (20) have been isolated from aborted fetuses. Important to expanding opportunities to conduct research in buffalo, information was obtained in the present study showing flow cytometry can be used with mAbs directly and indirectly labeled with fluorochromes. In addition data were obtained showing infection with BVDV leads to transient leukopenia similar to infection in cattle and fetal development. The availability of the large set of cross-reactivity mAbs now affords opportunity to expand research on the immune response to pathogens and vaccine development in buffalo.

## Materials and Methods

### Animals and Experimental Design

The experimental protocol for the study was authorized by the Ministry of Health (authorization n. 503/2017-PR) on the basis of Legislative Decree 26/03/14 on the protection of animals used for scientific purpose. The authorization limited the study to use of four healthy Mediterranean buffalo cows (6 years of age), negative for BVDV antibodies and antigens. Two cows were infected at 81 days (trial 1), the second two cows were infected at 203 days (trial 2) after artificial insemination. The pregnancy status was determined by clinical examination. During the infection trials, the animal health status was observed daily for a month after infection and then every 3 days until 15 days after calving.

### Virus

A non-cytopathic (ncp) field strain of BVDV-1 was used for experimental infection. Virus used in the study were prepared from the second passage in bovine kidney cells (MDBK). All animals were infected with a field type of BVDV-1. The virus was given by intranasal route at a dose of 2.5 mL × 10^5.00^ TCID_50_/mL for each animal.

### Sample Collection

Peripheral blood was collected from jugular vein into vacutainer tubes containing K_3_-EDTA, Li-Heparin and anticoagulant-free tubes were used for hematological, flow cytometric and serological analyses, respectively (Becton Dickinson, Plymouth, UK). Samples were collected at: 0, 3, 4, 6, 8, 10, 14, 17, 20, and 27 days post-infection (dpi). Nasal and vaginal swabs were taken at after day 10 dpi. Blood and ear notch samples were collected from calves immediately after birth and tested for presence of BVDV antigen and antibody.

### Hematological, Serological, and Molecular Analyses

Total and differential leukocyte counts were performed with a Cell Dyn 3700 (Abbott, Abbott Park, IL, USA) according to standard operation procedures. All serum samples were tested for BVDV antibodies and antigen by the commercial ELISAs ID Screen® BVD p80 Antibody Competition (IDvet, Grabels, France) and Bovine Viral Diarrhea Virus BVDV antigen test kit/serum plus (IDEXX, Westbrook, ME, USA), following the manufacturer's instructions. EDTA blood samples, nasal and vaginal swabs and ear notch samples were used for molecular analysis. Total RNA was extracted using the QIAampViral RNA mini Kit (Thermo Fisher Scientific, Waltham, MA, USA) as instructed by the manufacturer. The tests performed were an end point PCR that amplifies the 5'-UTR region using the pair of 324/326 primers amplifying a fragment of 288 bp (21) and the real-time RT-PCR described in the OIE Manual (22).

### Flow Cytometry: Monoclonal Antibodies and Labeling of Cells

The mAbs used in the present study are listed in Table 1. In a pre-trial study, we assessed the cell viability by LIVE/DEAD Fixable Near-IR stain kit **(**Thermo Fisher Scientific, Waltham, MA, USA). Since the viable cells were always higher than 95%, live/dead staining was omitted in trials 1 and 2. In the same study we compared the labeling efficiency of anti-human and anti-bovine mAbs cross-reactive with buffalo river type LMD orthologs (9, 10) including anti-bovine CD4 clone CC8 cross-reactive with buffalo swamp type (23). Flow cytometric analyses were performed: in the first trial at 0, 3, 4, and 14 dpi in the second trial at 0, 3, 4, 6, 8, 10, 14, and 17 dpi. In trial 1 we used a direct labeling method with purified mAbs labeled in house: clone IL-A11a using Zenon™ R-Phycoerythrin Mouse (Thermo Fisher Scientific, Waltham, MA, USA), MM1a and GB21a clones using Lightning-Link® APC (Allophycocyanin) and PE-Cy7 Conjugation Kit (Abcam, Cambridge, UK) respectively, following the manufacturer's instructions.

**Table 1:** Multicolor flow cytometric combinations used in this study.

**Panel-method**	**Antigen**	**Clone**	**Isotype**	**Source**	**Labeling**	**References**
1-indirect	CD8	7C2B	IgG_2a_	WSU-MAC	Fitc	(10)
	CD4	GC50A	IgM	WSU-MAC	PE	(10)
	δ chain	GB21A	IgG_2b_	WSU-MAC	PE-Cy7	(10)
	CD3	MM1a	IgG_1_	WSU-MAC	APC	(10)
2-indirect	CD25	LCTB2A	IgG_3_	WSU-MAC	Fitc	(10)
	ACT16	GB110a	IgM	WSU-MAC	PE	(10)
	CD4	IL-A11a	IgG_2a_	WSU-MAC	PE-Cy7	(10)
	CD3	MM1a	IgG_1_	WSU-MAC	APC	(10)
3-indirect	CD25	LCTB2A	IgG_3_	WSU-MAC	Fitc	(10)
	ACT16	GB110a	IgM	WSU-MAC	PE	(10)
	CD8	7C2B	IgG_2a_	WSU-MAC	PE-Cy7	(10)
	CD3	MM1a	IgG_1_	WSU-MAC	APC	(10)
4-indirect	CD25	LCTB2A	IgG_3_	WSU-MAC	Fitc	(10)
	δ chain	GB21A	IgG_2b_	WSU-MAC	PE	(10)
	CD8	7C2B	IgG_2a_	WSU-MAC	PE-Cy7	(10)
	CD335	AKS1	IgG_1_	Bio-Rad	APC	(10)
5-indirect	CD25	LCTB2A	IgG_3_	WSU-MAC	Fitc	(10)
	CD209	209MD26A	IgG_2a_	WSU-MAC	PE	(10)
	CD14	CAM66A	IgM	WSU-MAC	PE-Cy7	(10)
	CD21	GB25A	IgG_1_	WSU-MAC	APC	(10)
6-direct	CD8	CC63	IgG_2a_	Bio-Rad	Fitc	(9)
	CD21	CC21	IgG_1_	Bio-Rad	Fitc	(9)
	CD4	IL-A11a	IgG_2a_	WSU-MAC	PE	(10)
	CD335	AKS1	IgG_1_	Bio-Rad	PE	(10)
	CD14	TÜK4	IgG_2a_	Bio-Rad	PE-Cy5.5	(9)
	WC1-N2	BAQ4A	IgG_1_	WSU-MAC	PE-Cy7	(10)
	CD3	MM1a	IgG_1_	WSU-MAC	APC	(10)

*WSU-MAC = Washington State University-Monoclonal Antibody Center (Pullman. WA, USA); Bio-Rad Laboratories (Hercules, CA, USA). The anti-bovine CD4-PE conjugated (clone CC8, Bio-Rad), since negative, was not included in panel 6*.

**Figure 1:**
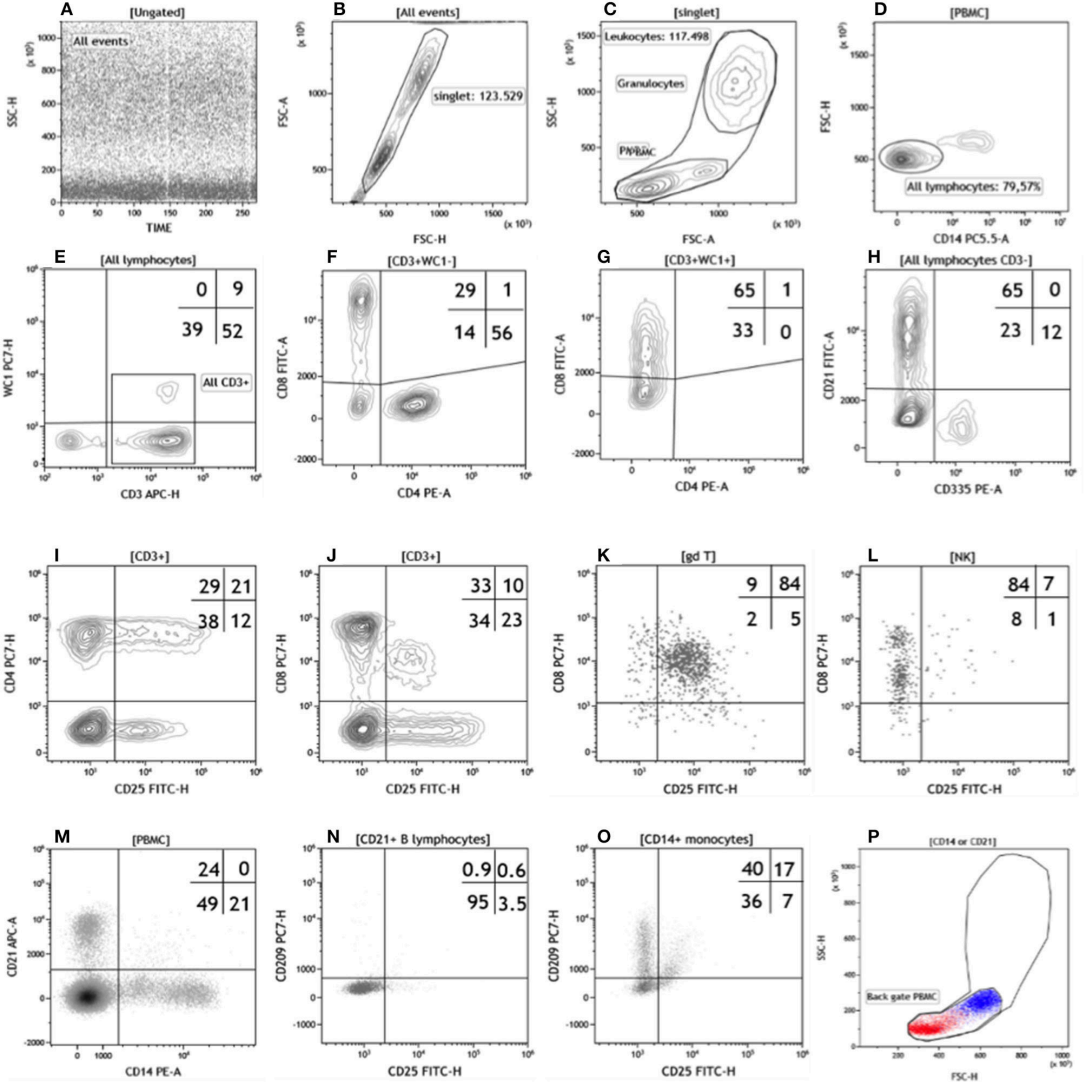
Flow cytometric gating strategy used in this study. The gating strategy **(A–H)** used to identify the lymphocyte subsets in Trial 1 was: **(A)** time parameter vs. SSC or FL to exclude event burst, **(B)** FSC-H vs. FSC-A to exclude doublets, **(C)** FSC vs. SSC to exclude debris, damaged and/or dead cells, then identify total leukocytes and PBMC, **(D)** CD14 vs. WC1 to identify total T lymphocytes and the WC1 subset. To characterize the other T lymphocyte subsets we used CD4 vs. CD8 for both WC1^−^CD3^+^ **(F)** and WC1^+^CD3^+^ **(G)** T cells. In buffalo the CD8 antigen is expressed by most γδ T lymphocytes (10), then we calculated the absolute value of CD3^+^CD8^+^ (Table 3 and Supplementary Table 1) as the total of upper left quadrant events in **(F,G)** dot plots. All lymphocytes CD3^−^ (bottom left in the dot plot **E**) were used to identify CD21^+^ B lymphocytes (top left) and NK cells (CD335^+^ bottom right) in the **(H)** dot plot. The gating strategy **(I–O)** used in Trail 2 had in common the first three gates (not shown) of Trail 1 **(A–C)**. For Panel 1, a CD3 vs. δ chain CD4 or CD8 dot plots were used to identify T lymphocyte subsets (not shown). In the Panel 2 and 3 we used a CD3 vs. SSC dot plot to identify all CD3 positive lymphocytes while CD3 vs. CD4 or CD8 dot plots to identify CD4 or CD8 T subsets, respectively (not shown). In the dot plot CD25 vs. CD4 **(I)** or CD8 **(J)**, the CD3^+^ gate was used to evaluate the percentage of CD4 or CD8 expressing CD25. In Panel 4, the δ chain vs CD335 dot plot was used to identify γδ T lymphocytes and NK cells (not shown) and then differentiated in the CD25 vs. CD8 dot plot for the co-expression of the two antigens (**K,L**, respectively). In Panel 5, the PBMC gate was used to identify CD21^+^ B lymphocytes and CD14^+^ monocytes **(M)**, which were subsequently characterized by co-expression of CD25 and CD209 antigens (**N,O**, respectively). Finally back gating on dot plot FSC vs. SSC confirmed the validity of the gating strategy **(P)**. For a better separation of cell clusters, we used the most appropriate combinations of fluorescent parameters (Hight-H and Area-A). For a better visualization we show the results as density dot plot **(A,K–O)**, contour plots **(B–J)**, and color dot plot **(P)** with a blue (CD14^+^ monocytes) and red (CD21^+^ B lymphocytes) color coding.

Fifty microliters of whole heparinized blood sample were incubated with saturating concentrations of mAbs in a final volume of 100 μL with phosphate buffered saline (PBS, pH 7.2) for 15 min at 4°C in the dark. Following incubation, erythrocytes were lysed with 1.0 mL of Tris-buffered ammonium chloride solution (0.87% w/v, pH 7.3) for 10 min, then mixed with 2 mL of cold PBS and then centrifuged at 300 g for 5 min. Pellets of labeled cells were resuspended in 150 μL of cold PBS for flow cytometric analysis. In trial 2 we used mAbs indirectly labeled with fluorochrome conjugated goat isotype specific anti-mouse antibodies. In brief, 2.0 mL of anticoagulated whole blood was added to a 50 mL polypropylene tube and lysed with 20.0 mL of the same ammonium chloride solution. The cells were sedimented by centrifugation and then resuspended in 25 mL of cold PBS. 5 × 10^5^ cells were incubated with 300 ng of each mAb in a final volume of 100 μL of PBS for 15 min at 4°C in the dark. Cells were washed (300 g for 5 min) then incubated under the same conditions using goat anti-mouse isotype specific secondary antibodies at optimal dilutions. Following incubation for 15 min, the cells were washed in PBS. The cells were resuspended in 150 μL for flow cytometric analysis. Fluorescence Minus One (FMO) controls were used to the correct quadrant positions for CD8, CD14, CD21, CD25, and CD209 molecules. A CytoFLEX flow cytometer (Beckman Coulter, Brea, CA, USA) was used to collect at least 5 × 10^4^ leukocytes. Data were analyzed using Kaluza software v. 2.1 (Beckman Coulter, Brea, CA, USA). For each lymphocyte subset, we applied the specific gating strategy (Figure 1), calculating the percentage of the subsets on total lymphocytes obtained in the forward scatter (FSC) *vs*. side scatter (SSC) dot plot. The relative percentages were then used to estimate the absolute value of the subset using the absolute values produced by Cell Dyn.

### Statistical Analyses

For each hematological and cytometric variable, variation time dependent was investigated by fitting the following mixed linear model with the MIXED procedure of SAS/STAT 9.2 (SAS Institute Inc., Cary, NC, USA) (24):

$$\begin{array}{l} \text{Y}=\mu +\text{TRIAL}+\text{DPI}+\text{TRIA}{\text{L}}^{*}\text{DPI}+\text{c}+\text{e} \end{array}$$

where y = hematological or cytometric parameters; μ =overall mean; TRIAL = fixed effect of the infection (two classes: 1, 2); DPI = fixed effect of the days post infection (10 classes: 0, 3, 4, 6, 8, 10, 14, 17, 20, and 27 for hematological and four classes: 0, 3, 4, and 14 for cytometric parameters respectively); c = random effects of individual buffalo cow; e = random residual.

## Results

### Clinical Observations

The experimentally infected animals showed no change in rectal temperature, signs or clinical symptoms. Later, however, trial 1 animals presented severe clinical complications: Buffalo-1 aborted 57 days after infection (138 days of gestation), while Buffalo-2 presented prolapse the day after calving and died.

### Serological and Molecular Results

All animals showed seroconversion, between 14 and 27 dpi, and all calves born from these buffaloes had BVDV antibodies and were negative for viral antigen, except the calf born from Buffalo-2 (trial 1) which was negative for both. Except for a trial 2 animal, PCR was positive at 3, 6 (nasal and vaginal swabs), and 8 dpi (nasal swap), blood samples, nasal, and vaginal swabs from other animals were negative for viral RNA.

### Hematological Analyses

As summarized in Table 2 and in Supplementary Figure 1, the experimental infection caused marked leukopenia due to a statistically significant lymphopenia. All animals showed the same trend: a rapid diminution in lymphocytes first evident at 3 dpi, with low values up to 8 dpi, then an increase at 10 dpi. A subsequent decrease occurred from 14 to 27 dpi, although the differences were not statistically significant. The other leukocyte subpopulations did not show significant changes or evident trends post-infection due to a marked variability between animals. In trial 1, the infection effects were more evident. Lymphopenia reached higher levels (−66 vs. −50%) than trial 2 (Supplementary Table 1), and the increase shown at 10 dpi respect to 0 dpi was more pronounced in the second than first infection trial (+78 vs. −1%).

**Table 2:** Comparison of total and differential leukocyte absolute counts (10^9^ cells/L) of infected buffaloes.

	**dpi**	**WBC**		**Neutrophils**		**Lymphocytes**		**Monocytes**		**Eosinophils**		**Basophils**	
		**Mean**	**SD**	**Mean**	**SD**	**Mean**	**SD**	**Mean**	**SD**	**Mean**	**SD**	**Mean**	**SD**
Trial 1 (*N* = 2)	0	6.29	0.33	2.66	0.42	2.87	0.59	0.55	0.09	0.17	0.07	0.04	0.00
	3	5.15	0.21	3.11	0.65	1.08	0.58	0.65	0.01	0.28	0.16	0.03	0.02
	4	3.54	0.62	1.83	0.54	1.00	0.19	0.48	0.05	0.17	0.24	0.06	0.07
	6	4.16	1.05	2.17	0.87	0.97	0.12	0.89	0.10	0.10	0.02	0.03	0.02
	8	4.80	0.51	2.45	0.02	1.35	0.62	0.90	0.19	0.01	0.00	0.10	0.05
	10	6.31	0.93	2.46	0.79	2.83	0.22	0.70	0.26	0.29	0.07	0.02	0.02
	14	6.34	0.31	3.23	0.76	2.06	0.11	0.84	0.31	0.19	0.02	0.03	0.02
	17	7.94	1.39	4.78	0.72	2.34	0.54	0.65	0.13	0.01	0.00	0.17	0.01
	20	6.66	1.23	3.12	0.54	2.58	0.64	0.69	0.13	0.23	0.12	0.05	0.02
	27	6.77	0.88	3.03	0.06	2.91	1.02	0.45	0.03	0.34	0.04	0.03	0.00
Trial 2 (*N* = 2)	0	7.27	2.43	3.89	1.91	2.16	0.39	0.83	0.20	0.31	0.43	0.09	0.10
	3	5.61	0.59	3.28	0.49	1.38	0.08	0.66	0.29	0.26	0.31	0.06	0.06
	4	5.43	0.44	3.27	0.49	1.12	0.01	0.87	0.03	0.00	0.00	0.17	0.11
	6	5.28	0.05	3.21	0.08	1.15	0.13	0.71	0.15	0.21	0.15	0.04	0.05
	8	5.20	0.16	3.13	0.01	1.08	0.20	0.87	0.03	0.09	0.08	0.05	0.04
	10	8.02	1.61	2.79	0.64	3.85	0.83	0.86	0.09	0.50	0.05	0.01	0.00
	14	6.60	1.73	3.19	1.06	2.30	0.16	0.95	0.49	0.01	0.01	0.15	0.02
	17	6.65	0.54	4.30	0.38	1.57	0.24	0.67	0.17	0.00	0.00	0.09	0.06
	20	7.24	0.98	4.71	0.71	1.81	0.07	0.55	0.16	0.14	0.15	0.04	0.01
	27	6.80	1.77	3.06	0.73	2.58	0.98	0.90	0.09	0.18	0.24	0.09	0.10
Mean infection (*N* = 4)	0	6.78^a^	1.53	3.27	1.33	2.51^A^	0.58	0.69	0.20	0.24	0.27	0.07	0.06
	3	5.38^a^	0.45	3.19	0.48	1.23^B^	0.38	0.66	0.17	0.27	0.20	0.04	0.04
	4	4.48^b^	1.18	2.55	0.94	1.06^B^	0.13	0.68	0.23	0.09	0.17	0.11	0.10
	6	4.72^a^	0.89	2.69	0.79	1.06^B^	0.14	0.80	0.15	0.16	0.11	0.04	0.03
	8	5.00^a^	0.38	2.79	0.40	1.22^B^	0.41	0.88	0.11	0.05	0.06	0.07	0.05
	10	7.16^a^	1.46	2.63	0.62	3.34^A^	0.77	0.78	0.18	0.40	0.13	0.02	0.01
	14	6.47^a^	1.03	3.21	0.75	2.18^A^	0.18	0.90	0.34	0.10	0.10	0.09	0.07
	17	7.30^a^	1.14	4.54	0.55	1.95^A^	0.56	0.66	0.12	0.00	0.00	0.13	0.06
	20	6.95^a^	0.97	3.91	1.05	2.19^A^	0.58	0.62	0.14	0.18	0.12	0.05	0.02
	27	6.78^a^	1.14	3.04	0.42	2.74^A^	0.84	0.68	0.27	0.26	0.17	0.06	0.07

*Significance test was not performed for trials 1 and 2 due to the small number of animals. Significant differences are referred to individual classes of leukocytes (column) and considered between 0 dpi and single time points (e.g., 0 vs. 3 dpi)*,

A,B *P < 0.01*;

a,b *P < 0.05*.

*WBC, white blood cells (total leukocytes); SD, standard deviation*.

### Antibody Labeling Evaluation by Flow Cytometry

All monoclonal antibodies used in trials 1 and 2 (Table 1) showed comparable labeling efficiency evaluated by percentage of positive cells. Unexpectedly the CC8 clone anti-bovine CD4 was negative, although it had been reported as positive swamp water buffaloes (23).

### Flow Cytometric Evaluation of Lymphocyte Subset Alterations

Statistical assessments were done using time points 0, 3, 4, 14 dpi common to both trials. The absolute values and percentage of differences between pre- and post-infection values are summarized in Table 3 and supplementary Table 1. Since the pre-infection absolute values of lymphocyte subsets, excluding B cells, obtained by direct (trial 1) or indirect labeling (trial 2) methods were similar, we considered the two flow cytometric approaches comparable. On average, the two infections produced severe lymphopenia due to the general decrease of all lymphocyte subsets (Table 3). T lymphocytes (CD3^+^) decreased significantly by 3 and 4 dpi (*P* < 0.01) with a maximum decrease of −55.8%. At 14 dpi, the absolute values were not statistically different from 0 dpi. CD4^+^ and CD8^+^ T cells had a similar dynamic: they decreased by 53.4% (*P* = 0.02) and 56.7% (*P* = 0.02), respectively, and both reached the minimum at 4 dpi. At 14 days while CD4^+^ T lymphocytes had the same values as the pre-infection, CD8^+^ T lymphocytes were still −13.6% compared to 0 dpi. The average CD4:CD8 ratio did not change significantly. Statistically significant differences were observed in other cell subsets, CD21^+^ B lymphocytes (*P* = 0.04) and NK cells (CD335^+^) (*P* < 0.001). Maximum average decrease (−71.5%) was shown by NK cells at 4 dpi. The absolute number of lymphocyte subsets indicated clearly that the lymphopenia caused by the infection was more pronounced when the animals were infected at 81 days post insemination compared to 203 days of gestation. At 3 dpi, the total CD3^+^, CD4^+^, and CD8^+^ lymphocytes decreased by 77.1, 83.8, and 65.4% in trial 1 compared to −22.2, −21.9, and −41.5% in trial 2, respectively. In addition, at 14 dpi, trial 1 animals did not return to their pre-infection values (−30.7, −21.7, and −38.5%) while in trial 2 animals increased by +13.5, +20.4, and +5.8%. The CD4:CD8 ratio showed a clear divergence between the two groups. While in trial 1 the effect of the infection was evident in reduction in the ratio from 1.9 at 0 dpi to 0.9 at 3 dpi (−52.6%), a positive increase to 35.7% was observed in trial 2 (Table 3). CD209 expression (trial 2) was always negative on CD21^+^ B lymphocytes, whereas CD14^+^ monocytes showed an evident increase from 8 to 17 dpi compared to baseline. At the same time point, we identified a small cellular subset with a phenotype CD21^−^CD14^−^CD209^+^ (Supplementary Figure 2).

**Table 3:** Comparison of absolute counts of lymphocyte subsets evaluated by flow cytometry (10^9^ cells/L).

		**0 Dpi**		**3 Dpi**		**4 Dpi**		**14 Dpi**	
		**Mean**	**SD**	**Mean**	**SD**	**Mean**	**SD**	**Mean**	**SD**
Trial 1 (*N* = 2)	CD3^+^	1.78	0.43	0.41	0.13	0.55	0.07	1.23	0.04
	CD21^+^ B	0.90	0.13	0.58	0.42	0.32	0.05	0.61	0.10
	NK (CD335^+^)	0.19	0.03	0.09	0.03	0.05	0.02	0.18	0.00
	CD3^+^WC1^+^	0.26	0.07	0.15	0.06	0.14	0.02	0.15	0.01
	CD3^+^CD4^+^	0.99	0.13	0.16	0.08	0.35	0.11	0.78	0.03
	CD3^+^CD8^+^	0.52	0.20	0.18	0.04	0.19	0.02	0.32	0.03
	CD4:CD8	1.9	0.6	0.9	0.1	2.4	1.2	2.4	0.4
Trial 2 (*N* = 2)	CD3^+^	1.58	0.16	1.23	0.11	0.93	0.04	1.79	0.34
	CD21^+^ B	0.41	0.21	0.11	0.03	0.14	0.07	0.37	0.25
	NK (CD335^+^)	0.17	0.03	0.04	0.00	0.05	0.02	0.13	0.07
	CD3^+^ γδ T	0.26	0.12	0.15	0.02	0.13	0.01	0.23	0.02
	CD3^+^CD4^+^	0.83	0.17	0.73	0.00	0.55	0.07	1.12	0.22
	CD3^+^CD8^+^	0.57	0.01	0.39	0.08	0.37	0.10	0.70	0.20
	CD4:CD8	1.4	0.3	1.9	0.41	1.5	0.62	1.6	0.15
Mean infection (*N* = 4)	CD3^+^	1.68^A^	0.29	0.82^B^	0.48	0.74^B^	0.22	1.51^A^	0.38
	CD21^+^ B	0.66^a^	0.32	0.34^a^	0.36	0.23^b^	0.11	0.49^a^	0.21
	NK (CD335^+^)	0.18^A^	0.03	0.06^B^	0.04	0.05^B^	0.02	0.16^A^	0.05
	CD3^+^CD4^+^	0.96^a^^A^	0.13	0.45^b^	0.33	0.45^B^	0.14	0.95^a^	0.23
	CD3^+^CD8^+^	0.59^a^^A^	0.14	0.28^b^	0.13	0.26^B^	0.14	0.51^a^	0.25
	CD4:CD8	1.71	0.50	1.39	0.67	2.05	0.95	2.03	0.53

*Trial 1 values were obtained from multicolor flow cytometry panel 6, trail 2 values from panels 1–5 (Table 1). Significance test was not performed for trials 1 and 2 due to the small number of animals. Significant differences are referred to single lymphocytes subset (line) between pre- and post-infection time points: 0 vs. 3 dpi; 0 vs. 4 dpi; 0 vs. 14 dpi*.

A,B *P < 0.01*;

a,b *P < 0.05*.

### Flow Cytometric Evaluation of Cell Activation Status

In trial 2 we evaluated the cell activation status by CD25 and ACT16 antigen expressions. CD25 (IL-2 receptor) was expressed on 79% of γδ, 38% of CD4^+^, and 35% of CD8^+^ T lymphocytes, 23% of CD14^+^ monocytes, 8% of CD21^+^ B lymphocytes, and 7% of NK cells. The only difference noted in expression after infection was on all T lymphocytes (CD3^+^) 8 dpi (+33%). ACT16, a molecule upregulated on *in vitro* activated CD4^+^ and CD8^+^ T lymphocytes (10), was never expressed in this pilot trial.

## Discussion

Flow cytometry is being used extensively in the study of the immune response to pathogens in humans and some economically important livestock species. A few mAbs specific for bovine LDM, however, are available conjugated to fluorochromes and was therefore useful to identify some cross-reactive anti-human mAbs. Although considerable progress has been made in identifying mAbs for use in buffalo, there is still a need to expand the mAb reagents for use in this species. In the present study we tested the CC8 anti-bovine CD4 mAb that, although cross-reactive with swamp buffalo (23), was negative with river-type buffalo. In cattle we noted that missense mutations Q306H and K310N in exon 5 of CD4 gene prevented the binding of the CC8 mAb (25). In an *in silico* study (unpublished), we verified that the genomic sequences of the river-type buffalo the L-glutamine 306 is conserved, but two Lysines (308 and 310) are changed to Asparagine and Serine, respectively. Although swamp-type buffalo exon 5 sequences are not published, this labeling difference could be an indirect confirmation that the same mutations in river buffalo account for loss of the epitope identified by CC8. In buffalo, moreover, flow cytometric methods are still under development. One of the current issues has been whether there is any difference in the use of mAbs directly or indirectly labeled with fluorochromes. Most of the mAbs for research in livestock are only available unconjugated with fluorochromes. For profit companies have developed three methods for labeling mAbs with fluorochromes: (1) goat fluorochrome polyclonal and isotype specific anti-mouse antibodies for use in indirectly labeling mAbs; (2) Zenon fluorochrome conjugated isotype specific Fab for binding to mAbs immediately for use in multicolor flow cytometry; (3) kits for in house directly conjugating mAbs with fluorochromes. Directly fluorochrome conjugated anti-human and anti-bovine LDMs, with potential cross-reactivity with buffalo and mAbs directly conjugated with fluorochromes with commercial kits were used to compare use in multicolor flow cytometry. No difference was observed in the methods for multicolor flow cytometry. Indirect labeling can be used with many of the initial multicolor flow cytometric studies in buffalo minimizing the cost of reagents. Zenon second step reagents can be used where there is a need to compare labeling with mAbs of the same isotype. The kit prices are still high and add considerably to the cost multicolor flow cytometry. Where essential, kits are available for directly conjugating mAbs.

A second issue for introducing flow cytometry to the study of the immune response to pathogens in buffalo has been when and how to initiate investigations. Initial comparative studies of the buffalo immune system with cattle have shown there are similarities and differences that need to be taken into consideration when studying the immune response to common pathogens (10). We selected BVDV as a model pathogen to initiate use of flow cytometry in the study of infectious diseases in buffalo because of ongoing interest in the pathogenesis BVDV in cattle (6) and evidence that it is an important pathogen for buffalo also. We used the study to compare direct and indirect labeling techniques and gain comparative information on the effect of infection on the composition of leukocyte subsets in blood during the 1st days of infection. The simultaneous use of FCM and routine hematological analysis revealed a similar modulation of the lymphocyte frequency caused by infection with ncp BVDV-1 as in other species (6, 26). We observed a significant reduction of leukocytes, by 4 dpi, and of total lymphocytes from 3 dpi to 8 dpi; no significant variations were observed in other leukocyte subpopulations (Table 2). The results were similar to results reported in bovine and alpaca species (6, 26) with a significant reduction of all CD3^+^, CD4^+^, and CD8^+^ T lymphocytes, CD21^+^ B lymphocytes and NK cells at 3 and 4 dpi. No variation was observed in CD4:CD8 ratio. Our results are in accordance with Ellis et al. (6) that showed a significant decrease of WBC and lymphocytes (CD2^+^, CD4^+^, and CD8^+^) in bovine peripheral blood at 3, 5, and 7 dpi with ncp BVDV. They also observed no significant variations in the CD4:CD8 ratio, which can be explained by an equal reduction in both subsets. Similarly, Gånheim et al. (29) reported that the total leukocytes, neutrophils and lymphocytes decreased significantly from day 3 from inoculation of bovine calves with BVD virus and then increased at the 9 dpi. Reduction in the frequency of CD8^+^ and WC1^+^ cells was observed on days 8, 11.5, and 15.5 of the study and 11.5, 15.5, and 22.5 respectively, compared to day 2.5 of the study. No differences were observed in the numbers of B lymphocytes.

CD25, a subunit of the IL-2 receptor (IL-2R), is expressed on activated cells including T lymphocytes, B lymphocytes, and monocytes. In trial 2 the percentage of cells CD3^+^/CD25^+^ showed an increase (+33%) at 8 dpi (data not shown). A significant increase in activated lymphocytes has been reported in alpacas experimentally infected at 9 dpi (26). These observations might suggest that buffaloes and alpacas, two species thought to be more resistant to BVDV, could have a stronger lymphocyte activation than in cattle (27). Comparative studies can now be conducted to confirm and extend studies to elucidate differences in the immune response to BVDV.

Brock and coworkers (28) reported that the outcome of fetal infection was dependent on the stage of gestation. In our study, although all animals did not show clear clinical signs and symptoms (e.g., diarrhea, respiratory problems, and fever), trial 1 animals incurred severe clinical complications. Buffalo-1 aborted 57 days after the infection (138 days of gestation); unfortunately, it was not possible to perform necropsy on the aborted fetus. We could not conclude that the abortion was due to the infection. Buffalo-2 died of a postpartum prolapse and her calf died 10 days after calving. Fetal death and abortion occur during BVDV infection in cattle (29) and buffalo (20). The prolapse could be the result of increased susceptibility to bacterial infections associated with BVDV infection (30). In trial 1, our results showed a more pronounced and prolonged leuko- and lymphopenic post-infection effect that might account for the observed effects (Table 2). However, more extensive studies are needed to follow up this possibility.

In conclusion, this preliminary study shows that the availability of an extensive set of mAbs in buffalo has increased opportunities to conduct studies on the immune response to pathogens and development of vaccines. Moreover, since in buffalo many pathological conditions such as BVD, are asymptomatic, the flow cytometric approach may be helpful in the diagnosis and/or prognosis of some conditions.

## Data Availability Statement

The datasets generated for this study are available on request to the corresponding author.

## Ethics Statement

The animal study was reviewed and approved by Ministry of Health (authorization n. 503/2017-PR).

## Author Contributions

ED: conceptualization. FG, AM, SP, FF, GD, and ED: methodology. FG, AM, SP, RS, AD, CC, MS, CG, and DV: sample collection and analysis. FG and WD: original draft preparation. AM, MS, GD, ED, FG, and WD: review and editing. All authors have read and agreed to the published version of the manuscript.

## Conflict of Interest

The authors declare that the research was conducted in the absence of any commercial or financial relationships that could be construed as a potential conflict of interest.
