# Supplementary figures and images for: Assessment of Multicolor Flow Cytometry Panels to Study Leukocyte Subset Alterations in Water Buffalo (Bubalus bubalis) During BVDV Acute Infection
Source: Front Vet Sci. 2020 Oct 16;7:574434. doi: 10.3389/fvets.2020.574434 (PMC7596219; doi:10.3389/fvets.2020.574434)

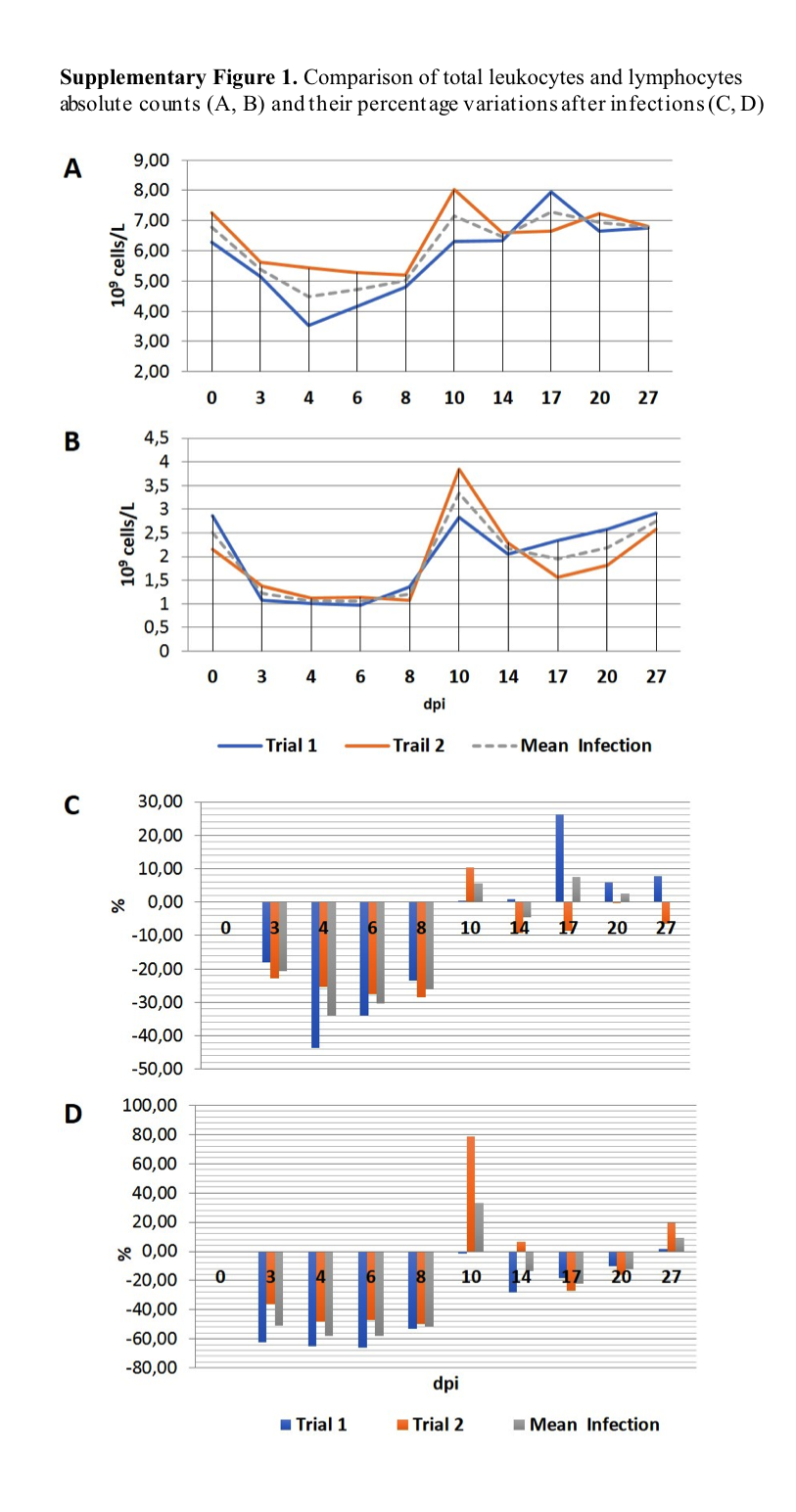

Supplement: Supplementary file 1 [file Image_1.tiff]
