# Supplementary material for: Assessment of Multicolor Flow Cytometry Panels to Study Leukocyte Subset Alterations in Water Buffalo (Bubalus bubalis) During BVDV Acute Infection
Source: Front Vet Sci. 2020 Oct 16;7:574434. doi: 10.3389/fvets.2020.574434 (PMC7596219; doi:10.3389/fvets.2020.574434)
Supplement: Supplementary file 3 [file Table_1.docx]

**Supplementary Table 1.** Comparison of infection effects. Percentage differences between pre- and post-infection absolute values of lymphocytes subsets evaluated by flow citometry (Table 3).

|  | **Dpi** | **TRIAL 1 (N = 2)** | **TRIAL 2 (N = 2)** | **MEAN INFECTION (N=4)** |
| --- | --- | --- | --- | --- |
| **CD3^+^** | **3** | -77.1 | -22.1 | -51.2 |
|  | **4** | -68.8 | -41.1 | -55.8 |
|  | **14** | -30.7 | 13.5 | -9.9 |
| **CD21^+^ B** | **3** | -35.6 | -73.2 | -47.5 |
|  | **4** | -64.4 | -65.1 | -65.1 |
|  | **14** | -32.2 | -9.8 | -25.5 |
| **NK (CD335^+^)** | **3** | -52.6 | -76.5 | -64.1 |
|  | **4** | -73.7 | -70.6 | -71.5 |
|  | **14** | -5.3 | -23.5 | -11.1 |
| **CD3^+^CD4^+^** | **3** | -83.8 | -21.9 | -53.3 |
|  | **4** | -64.6 | -40.9 | -53.4 |
|  | **14** | -21.7 | 20.4 | -1.6 |
| **CD3^+^CD8^+^** | **3** | -65.4 | -41.5 | -51.9 |
|  | **4** | -72.1 | -44.8 | -56.7 |
|  | **14** | -38.5 | 5.8 | -13.6 |
